# TESTS TO ASSESS SENSITIZATION TO *ASPERGILLUS FUMIGATUS* IN CYSTIC FIBROSIS

**DOI:** 10.1590/1984-0462/;2017;35;3;00003

**Published:** 2017-07-13

**Authors:** Simone Santana Aguiar, Neiva Damaceno, Wilma Carvalho Neves Forte

**Affiliations:** a Setor de Pneumologia do Departamento de Pediatria e Puericultura da Irmandade da Santa Casa de Misericórdia de São Paulo, SP, Brasil.; b Equipe Multidisciplinar do Serviço de Referência de Tratamento de Fibrose Cística do Setor de Pneumologia do Departamento de Pediatria e Puericultura da Irmandade da Santa Casa de Misericórdia de São Paulo, SP, Brasil.; c Departamento de Ciências Patológicas da Faculdade de Ciências Médicas da Santa Casa de São Paulo, SP, Brasil.

**Keywords:** Aspergillus fumigatus, Cystic fibrosis, Skin tests, IgE, Allergic bronchopulmonary aspergillosis (ABPA)

## Abstract

**Objective:**

To evaluate the results of the tests used to identify the IgE mediated sensitization to *Aspergillus fumigatus* in patients with cystic fibrosis.

**Methods:**

This is a cross-sectional descriptive study with a convenience sample of 86 patients diagnosed with cystic fibrosis in the Reference Service in Cystic Fibrosis at a tertiary teaching hospital. The following tests were performed to assess the sensitization to *A. fumigatus* in patients with cystic fibrosis: Total serum IgE, eosinophil count, fungus detection through oropharyngeal swab or sputum culture, serum-specific IgE, and immediate-type hypersensitivity (IgE) skin tests. We compared the results of the different tests performed.

**Results:**

In 33 (38.4%) patients with cystic fibrosis, with ages ranging from 1 to 33 years (median of 8 years), the IgE-mediated *A. fumigatus* sensitization test results were: in 16 patients, there was an increase in serum-specific IgE (>0.35 kU/L); in 23, skin tests were positive; and six had sensitization in both tests. We observed two patients with eosinophilia (>1,000 eosinophils/mm3) and seven with increasing total serum IgE (>1,000 IU/mL), all of whom obtained negative results in skin tests and had no IgE increase specific to *A. fumigatus*. *A. fumigatus* was not detected in oropharyngeal swabs and/or sputum culture of any patients.

**Conclusions:**

We conclude that, among the tests used to assess sensitization to *A. fumigatus* in cystic fibrosis patients, both serum-specific IgE and immediate-type hypersensitivity (IgE) skin tests are required. Serum eosinophilia and respiratory secretion culture were not essential in this study.

## RESUMO

**Objetivo:**

Avaliar os resultados dos exames utilizados para identificar a sensibilização IgE-mediada ao *Aspergillus fumigatus* em pacientes com fibrose cística.

**Métodos:**

Estudo transversal descritivo com amostra de conveniência de 86 pacientes com fibrose cística, acompanhados em Serviço de Referência de Fibrose Cística de hospital universitário terciário. Realizaram-se exames para avaliar sensibilização ao *A. fumigatus* em pacientes com fibrose cística: IgE sérica total, contagem de eosinófilos sanguíneos, identificação do fungo por *swab* de orofaringe ou por cultura de escarro, IgE sérica específica e testes cutâneos de hipersensibilidade imediata. Foram comparados os resultados dos diferentes exames realizados.

**Resultados:**

Em 33 (38,4%) pacientes com fibrose cística, com faixa etária de 1 a 33 anos (mediana de 8 anos), os resultados dos exames sobre sensibilização IgE mediada ao *A. fumigatus* foram: em 16 pacientes, aumento de IgE sérica específica (>0,35 kU/L); em 23, positividade aos testes cutâneos; e seis mostraram sensibilização a partir dos dois exames. Foram observados dois pacientes com eosinofilia (>1.000 eosinófilos/mm^3^) e sete com aumento de IgE sérica total (>1.000 UI/mL), sem que esses apresentassem positividade aos testes cutâneos ou aumento de IgE específica ao *A. fumigatus*. Em nenhum paciente foi isolado *A. fumigatus* no *swab* de orofaringe e/ou na cultura de escarro.

**Conclusões:**

Concluímos que, entre os exames para avaliar a sensibilização ao *A. fumigatus* na fibrose cística, são necessários os teste cutâneos de hipersensibilidade imediata e a dosagem de IgE sérica específica ao *A. fumigatus.* A eosinofilia sérica e a cultura de secreções respiratórias não foram essenciais neste estudo.

## INTRODUCTION

Cystic fibrosis (CF) is an autosomal recessive disorder with different phenotypes, more common among people of white ethnicity. It mainly affects the respiratory and digestive systems, the sweat glands and the male reproductive system. In CF, mucociliary clearance and bronchial mucous production are defective, and the mucous produced is thick and viscous. Consequently, lung diseases become the major cause of morbidity and mortality for patients with CF. CF is a chronic disease that has seen remarkable progress in prognosis in recent years, besides increased survival rates, especially through early diagnoses using after-birth screening tests and with an improved control of the patients’ pulmonary disease and nutrition.^1^ ^,^ ^2^ ^,^ ^3^ ^,^ ^4^ ^,^ ^5^ ^,^ ^6^ ^,^ ^7^ ^,^ ^8^ ^,^ ^9^

*Aspergillus fumigatus* may sensitize patients with CF, leading to IgE-mediated hypersensitivity and allergic bronchopulmonary aspergillosis (ABPA) with variable prevalence.^10^ ^,^ ^11^ Among patients with CF, ABPA presents clinical manifestations that are very similar to those observed in CF exacerbation, making it difficult to diagnose, and requiring further tests. The frequency of ABPA in patients with CF is variable, ranging between 1 and 15%. The higher prevalence found in a survey conducted by the *European Epidemiologic Registry of Cystic Fibrosis* (ERCF), in comparison to that observed by the *Epidemiologic Study of Cystic Fibrosis* (ESCF), may be attributed to ethnic variances and the different diagnostic criteria used; the European criteria are more comprehensive than the ones used in the US study.^11^ ^,^ ^12^ CF patients must be tested for ABPA due to its impact on prognosis, so that diagnosis and treatment can be performed early to avoid greater pulmonary damage.

The criteria for the diagnosis of ABPA in CF have been constantly reviewed. Currently, these criteria are based on clinical manifestations and complementary tests: a positive result for immediate skin hypersensitivity to *A. fumigatus*, increased total serum IgE and *A. fumigatus*-specific IgE and/or IgG, blood eosinophilia, oropharyngeal swab or sputum culture for the detection of *A. fumigatus*.^11^ ^,^ ^12^ It is recommended that these tests be performed every six months for all patients with CF, in an attempt to diagnose ABPA. However, at present there are no studies regarding the need to perform all tests to check for the patients’ sensitization to *A. fumigatus*, thus generating high costs to health care centers treating CF patients. In this context, the goal of this study was to evaluate the results of tests used to identify the IgE-mediated *Aspergillus fumigatus* sensitization in patients with cystic fibrosis.

## METHOD

The project was approved by the university’s Research Ethics Committee (Project 065/06). An Informed Consent Form was signed by patients and/or guardians prior to any procedure related to the study.

A six-month long cross-sectional descriptive study was conducted in 2013, with a convenience sample of CF patients enrolled in outpatient clinics of the Reference Service for the treatment of CF in a tertiary university hospital. Each patient was submitted only once to each test.

We evaluated 86 patients with confirmed diagnosis of CF using two sweat chloride tests, by the Gibson and Cooke standardized method:^5^ quantitative pilocarpine iontophoresis with results higher than 60 mmol/L and sweat mass equal to or higher than 0.075 g.

We used the complementary tests and values routinely used to detect *A. fumigatus* sensitization:

1. Total serum IgE through the enzyme immunoassay method (ELISA - *Enzyme Linked Immuno Sorbent Assay*), considered high when higher than 1,000 IU/mL.^13^
2. *A. fumigatus*-specific IgE assay, conducted through RAST using the *UniCAP* *100* (*Pharmacia Diagnostics*) enzyme immunofluorescence technique. Sensitization was detected for values equal to or higher than 0.35 kUA/L.^14^
3. *A. fumigatus* immediate hypersensitivity skin test, performed at the Allergy and Immune Disorders division in accordance to standards: either positive for values equal to or higher than 3 cm with positive histamine control, or higher than or equal to positive histamine control with negative saline solution control. We used the *International Pharmaceutical Immunology S.A. -* (IPI®, Spain) standardized extract^15^.
4. Automated eosinophil count, conducted at the Clinical Pathology Service. Eosinophilia was diagnosed for values higher than 1,000 eosinophils/mm^3^.
5. Oropharyngeal swab and/or sputum culture to detect *A. fumigatus,* conducted at the Clinical Pathology Service, positive for the presence of fungus.^16^

## RESULTS

**Figure 1:**
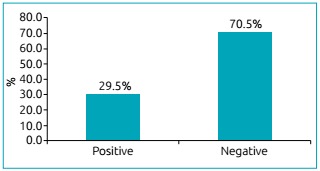
Results of skin tests of immediate hypersensitivity to *Aspergillus fumigatus* among the 78 Cystic Fibrosis patients (percentage of patients).

**Figure 2:**
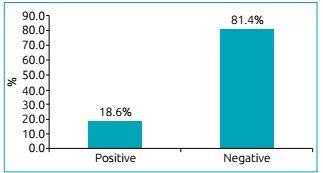
Positive sensitization to *Aspergillus fumigatus* by serum-specific IgE measurement determined by RAST (values ≥ 0.35 kU/L) among the 86 patients with Cystic Fibrosis (percentage of patients).

We analyzed 86 patients with CF, aged 1 to 33 years old, with median age of 8 years. The mean age was 10.8 ± 8.6 years (Table 1). Among the patients studied, 33 (38.4%) showed an increase in *A. fumigatus-*specific IgE through RAST (≥0.35 kU/L) and/or positive results on immediate hypersensitivity skin tests. Sixteen showed increase for serum-specific IgE (≥0.35 kU/L) and 23 obtained positive results on skin tests. Eight patients did not undergo skin tests due to difficulties in attendance. Among 33 patients, six were sensitive in two exams: Serum IgE and skin test (Table 2, Figures 1 and 2).

**Table 1:**
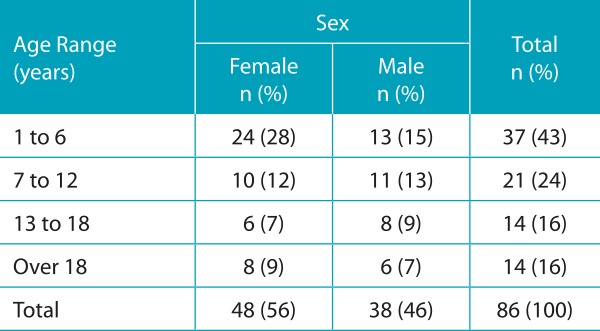
Distribution of patients with Cystic Fibrosis according to gender and age.

**Table 2:**
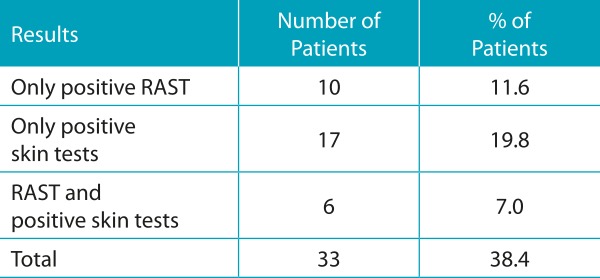
Distribution of patients with cystic fibrosis according to the results of serum specific IgE to *Aspergillus fumigatus* determined by RAST and skin tests of immediate hypersensitivity to *Aspergillus fumigatus*.

Eosinophilia (>1,000 eosinophils/mm^3^) was detected in two patients; total serum IgE was high (>1,000 IU/mL) in seven patients, and patients with eosinophilia were different from those with high total serum IgE. No patients’ oropharyngeal swab and/or sputum culture tested positive for *A. fumigatus*. Patients with eosinophilia or increased total IgE tested negative for *A. fumigatus*-specific sensitization skin tests and had no increase in *A. fumigatus-*specific serum IgE. ABPA was not detected in any of the patients followed.

## DISCUSSION

38.4% of patients with CF tested positive in the immediate hypersensitivity skin tests and/or presented an increase in *A. fumigatus*-specific serum IgE, exhibiting no changes in the other tests (total serum IgE, eosinophilia or presence of the fungus), thus not meeting the criteria for ABPA.

IgE-mediated hypersensitivity develops gradually with age and with the increased exposure to the allergen. Thus, in IgE-mediated reactions, sensitization to the specific allergen starts slow and builds up over time to the point in which one develops the disease. This is the case of well-known IgE-mediated diseases such as allergic rhinitis, allergic conjunctivitis, allergic asthma, food allergy and IgE-mediated latex allergy.^17^ Positive results are initially obtained in skin tests, and there is a slight increase in serum-specific IgE levels. With the continuous antigen exposure, serum-specific IgE levels increase, including values ≥ 3.5 kU/L, which indicates high sensitization. Even in this condition, there may not be an increase in total IgE levels or eosinophilia. When sensitization begins, clinical features are usually mild, a fact especially known in the case of latex allergy. Continuous exposure to allergens increases clinical features. ABPA IgE-mediated hypersensitivity seems to have the same pattern as other IgE-mediated reactions: patients initially present with low sensitization (≥0.35 kU/L), and later, with continuous exposure to the fungus, there may be changes in other tests and presence of clinical features.

The frequency of sensitization observed in this study is within the range of values reported in the literature for patients with CF sensitized to *A. fumigatus,* but negative for ABPA: 42% tested positive in skin tests, and 54% to increased serum-specific IgE levels.^18^ ABPA has been described more often in adolescents and adults with CF, as indicated by studies that show median age of 14.7 and 18 years of age.^19^ ^,^ ^20^ In the present study, as median age was 8 years old, it is possible that subjects were still developing sensitization to *A. fumigatus*; therefore, with age, they might develop ABPA.

The presence of *A. fumigatus* in lower airways pulls neutrophils into alveolar spaces, resulting in spore phagocytosis or the release of its granular contents, leading to fungal death. In cases of uncontrolled allergic asthma, research has shown changes in neutrophil activity, contributing with neutrophilia and worse allergy symptoms.^21^ Alveolar macrophages also play an important role in lung defense against *A. fumigatus*. The prolonged presence of fungi in the lungs results in diseases ranging from hypersensitivity (ABPA) to invasive cavitation secondary to spore germination.^22^ Thus, in order for ABPA to develop, an initial sensitization to fungi must take place, followed by their prolonged presence, just as in other IgE-mediated diseases.

In the present study, total serum IgE and peripheral blood eosinophil count increased in a small number of patients, who saw no rise in specific IgE levels and/or tested positive for the skin test. It is known that total serum IgE and eosinophil count may increase or remain normal in cases of IgE-mediated diseases, and that total IgE levels can be high even among individuals without IgE-mediated reactions. Total serum IgE results from the sum of one or more serum-specific IgE.^17^ Thus, in respiratory allergies, total serum IgE levels can be a result of the sum of serum IgE specific to the most common aeroallergens: *Dermatophagoides pteronyssinus,* *Dermatophagoides farinae* and *Blomia tropicalis*. Total serum IgE levels may be normal even among patients who are sensitive to all three mites, i.e., patients presenting with increased specific IgE levels corresponding to each of the three most common mites, thus causing respiratory allergies.^17^ The same should be true for *A. fumigatus* sensitization: Even though a specific *in vivo* IgE is high, this does not imply an increase of total IgE levels. In ABPA cases, sensitization to the fungus is higher, increasing specific IgE levels and a corresponding rise in total serum IgE.

The present study does not allow us to conclude which of the tests would be more adequate to check for *A. fumigatus* sensitization*:* *in vitro* or *in vivo* specific IgE. We believe both tests are complementary. A survey on *A. fumigatus* sensitization in patients with CF had 90% match for both exams.^23^ The literature also refers to the importance of setting IgE dosages specifically to *A. fumigatus* in CF cases.^24^

The literature further describes that even at an early age children may test positive for the immediate hypersensitivity skin test.^15^ This is relevant given the physiological immaturity of the inflammatory response, and the adaptive immune responses of young children. Immediate hypersensitivity skin tests indicate the presence of specific IgE attached to mast cells. In an initial contact with the allergen, the host activates type 2 T helper lymphocytes, which synthesize IL-4 and IL-13, cytokines that promote the differentiation of B lymphocytes into plasma cells that produce specific IgE. This IgE binds itself to mast cells containing receptors with high-affinity for IgE, thus sensitizing the cells.^17^ When the allergen is applied during the skin test, it reacts with the specific IgE attached to mast cells, thus releasing histamine from mast cells and forming wheals that lead to a positive test result. Previous studies have shown that, after six months, IgE-mediated skin tests already return positive.^15^ ^,^ ^25^ Research shows an increase in *A. fumigatus-*specific IgE as the lung disease caused by CF progress.^26^ This might occur with the patients in the present study.

In the follow-up of patients with CF, respiratory secretions are routinely collected. The results obtained here are likely owed to the short time frame of the study. In studies on the prevalence of ABPA, negative cultures are reported. Studies also highlight the fact that positive cultures have no diagnostic value. In addition, *A. fumigatus* is often isolated in lower airways of patients with CF with negative culture. The lack of effective mucociliary clearance leads to the accumulation of mucus, which is rich in nutrients for microorganisms, facilitating the spread and persistence of bacteria and fungi.^27^ ^,^ ^28^ ^,^ ^29^ These facts can explain the negative cultures observed in all patients studied.

The results of this study show that the tests indicating sensitization to *A*. *Fumigatus* are the immediate hypersensitivity skin test and the increase of serum-specific IgE. Assaying the eosinophil count in peripheral blood, total IgE serum levels and cultures to detect *A. fumigatus* is expensive and often difficult for patients, and may not test positive for *A.*fumigatus sensitization*.* The clinical manifestations of CF and ABPA overlap. Pulmonary infiltrates, bronchiectasis and chronic obstructive pulmonary disease are common in CF, resulting from repeated infections and diseases, regardless of the presence of ABPA. ABPA is possible when a clinical condition suffers acute or subacute worsening, with pulmonary exacerbations not attributable to another etiology and/or after failed attempts of treatment with antimicrobial agents against bacteria isolated in cultures. Under these conditions, it is recommended to check for the possibility of concomitant ABPA, considering the criteria proposed by the literature.^11^ ^,^ ^12^ ^,^ ^30^ ^,^ ^31^

The survival of patients with CF has increased, as shown by the records.^32^ If the financial resources addressed to the health of these patients can put to better use, as different sources have proposed, we will surely contribute with better quality of life for patients with CF.^33^

In order to assess sensitization to *A. fumigatus* in patients with CF, we conclude that two tests are necessary: the immediate hypersensitivity skin test and the determination of A.fumigatus-specific IgE levels*.* Eosinophilia detection, total serum IgE and swabs/cultures were not essential for the detection of *A. fumigatus* sensitization in young patients with CF. It is worth highlighting the limitations of this research, considering the number of patients and the relatively short time frame of the analysis. Even so, we demonstrated the importance of skin tests and of measuring serum IgE specific *A. fumigatus* to assess sensitization in cystic fibrosis patients.

In this context, we suggest that *in vitro* and *in vivo* tests for IgE specific to *A. fumigatus* be initially performed and, if sensitization is detected, other tests should be carried out: Total IgE levels, eosinophils count and fungus research, complying with the diagnostic criteria for ABPA in CF.
